# Lack of influence by endosymbiont *Wolbachia* on virus titer in the common bed bug, *Cimex lectularius*

**DOI:** 10.1186/s13071-019-3694-2

**Published:** 2019-09-09

**Authors:** Michael L. Fisher, Jay F. Levine, James S. Guy, Hiroyuki Mochizuki, Matthew Breen, Coby Schal, David W. Watson

**Affiliations:** 1 Department of Entomology and Plant Pathology, North Carolina State University, Raleigh, NC USA; 2 United States Navy Medical Service Corps, Raleigh, NC USA; 3 Department of Molecular Biomedical Sciences, North Carolina State University, Raleigh, NC USA; 4 Department of Marine, Earth and Atmospheric Sciences, North Carolina State University, Raleigh, NC USA; 5 Department of Population Health and Pathobiology, North Carolina State University, Raleigh, NC 27607 USA

**Keywords:** *Cimex lectularius*, *Wolbachia*, Endosymbiont, Cimicidae, Virus suppression, ssRNA

## Abstract

**Background:**

The common bed bug, *Cimex lectularius*, is an obligatory blood-feeding ectoparasite that requires a blood meal to molt and produce eggs. Their frequent biting to obtain blood meals and intimate association with humans increase the potential for disease transmission. However, despite more than 100 years of inquiry into bed bugs as potential disease vectors, they still have not been conclusively linked to any pathogen or disease. This ecological niche is extraordinarily rare, given that nearly every other blood-feeding arthropod is associated with some type of human or zoonotic disease. Bed bugs rely on the bacteria *Wolbachia* as an obligate endosymbiont to biosynthesize B vitamins, since they acquire a nutritionally deficient diet, but it is unknown if *Wolbachia* confers additional benefits to its bed bug host. In some insects, *Wolbachia* induces resistance to viruses such as Dengue, Chikungunya, West Nile, Drosophila C and Zika, and primes the insect immune system in other blood-feeding insects. *Wolbachia* might have evolved a similar role in its mutualistic association with the bed bug. In this study, we evaluated the influence of *Wolbachia* on virus replication within *C. lectularius*.

**Methods:**

We used feline calicivirus as a model pathogen. We fed 40 bed bugs from an established line of *Wolbachia*-cured and a line of *Wolbachia*-positive *C. lectularius* a virus-laden blood meal, and quantified the amount of virus over five time intervals post-feeding. The antibiotic rifampicin was used to cure bed bugs of *Wolbachia*.

**Results:**

There was a significant effect of time post-feeding, as the amount of virus declined by ~90% over 10 days in both groups, but no significant difference in virus titer was observed between the *Wolbachia*-positive and *Wolbachia*-cured groups.

**Conclusions:**

These findings suggest that other mechanisms are involved in virus suppression within bed bugs, independent of the influence of *Wolbachia*, and our conclusions underscore the need for future research.

## Background

The common bed bug, *Cimex lectularius*, is an obligate blood-feeding ectoparasite that has undergone a global resurgence in the last two decades (see reviews in [1]). Recent discoveries of resistance to diverse classes of insecticides [2–5] makes bed bug infestations increasingly difficult to control, but the significant fitness costs associated with resistance could be exploited in integrated pest management plans [6]. Highly specialized treatments which are cost-prohibitive to most low-income residents, coupled with insecticide resistance, exacerbate the resurgence by often failing to prevent reintroductions [7]. The profound resurgence of bed bugs in such frequent associations with humans and our domiciles could increase the threat of disease transmission.

Bed bugs, like the related triatomine bugs that transmit *Trypanosoma cruzi*, the etiological agent of Chagas disease, are hemimetabolous, so each instar and all adults require at least one blood meal to develop and reproduce. Such frequent re-feeding contaminates the indoor environment with histamine [8] and could contribute substantially to their importance as disease vectors. Yet, despite being exclusively hematophagous and intimately associated with humans, to date bed bugs have not been conclusively implicated in vector-borne disease transmission. Bed bugs can acquire a myriad of blood-borne pathogens from their hosts, but in the case of ingested viral particles, most viruses do not or are not capable of replicating once inside the bed bug (reviewed in [9–12]). Hepatitis B virus (HBV) is a notable exception, however. It has been detected > 45 days post-ingestion, after direct injection into the hemocoel, and HBV is stercorarially shed in bed bug feces, suggesting the possibility of mechanical transmission [10, 13, 14], if HBV can enter and replicate in the hemocoel. Recently, bed bugs have been shown to experimentally acquire, maintain, and effectively transmit *T. cruzi* [15], and *Bartonella quintana*, the etiological agent associated with Trench fever [16], but a survey of field-collected bed bugs failed to detect *Bartonella* [17]. However, the latter survey did detect *Burkholderia multivorans* in bed bugs.

Microbe-microbe interactions with respect to pathogen suppression have been studied in various blood-feeding insects such as the kissing bug *Rhodnius* [18, 19], tsetse fly *Glossina* [20, 21], and mosquitoes (reviewed in [22]), as well as in plant-feeding fruit flies and aphids. The insect microbiome can modulate vector competence of the host for arboviruses (reviewed in [21, 23, 24]), and these influences have been most often evaluated in associations of the endosymbiont *Wolbachia* with fruit flies and mosquitoes. *Wolbachia* protects *Drosophila* against virus-induced mortality for Drosophila C virus (DCV) and Flock house virus (FHV) [25, 26]. *Wolbachia* also stimulates immune gene expression in several mosquito species [27–31], and thus increases resistance to, and reduces the vector competence of *Aedes aegypti*, *Aedes albopictus* and *Culex quinquefasciatus* mosquitoes for viruses such as Dengue, Chikungunya, West Nile, and Zika [32–38]. Dengue, Chikungunya, Zika, West Nile, DCV, and FHV are all positive-sense single-stranded RNA viruses (+ssRNA), suggesting that the anti-viral effects induced by *Wolbachia* in *Drosophila* and mosquitoes might be limited to RNA viruses [39, 40].

*Cimex lectularius* harbors *Wolbachia* as its primary endosymbiotic nutritional mutualist that biosynthesizes B vitamins for its nutritionally deficient host [41, 42] in a co-dependent relationship that has presumably evolved over several million years [43]. Additional fitness benefits that the endosymbiont might confer upon bed bugs have not been investigated. Similar to the effects reported in other arthropods, *Wolbachia* could influence the vector competence of *C. lectularius* through mechanisms involving interactions with the host or with ingested pathogens, thus preventing certain viruses from replicating within the host. This may explain in part why bed bugs are not a major disease vector for arboviruses. The objective of this study was to compare virus titer in *Wolbachia*-positive and *Wolbachia*-free *C. lectularius* at several time intervals after ingesting a virus-laden blood meal.

## Methods

### Establishment of *Wolbachia*-free *C. lectularius* colonies

The Winston-Salem (WS) strain of *C. lectularius* was collected in Winston Salem, NC in 2008 and fed defibrinated rabbit blood (Hemostat Laboratories, Dixon, CA, USA) in an artificial feeding system, as described by Sierras & Schal [44]. Ten adult males and 20 adult females of the WS strain were divided equally and placed into two separate 20-ml glass vials with screened-caps and a creased section of card stock for harborage. These vials were then placed into a plastic container (17.8 × 12.7 × 10.2 cm) as additional protection against environmental bacteria. A semi-sterile incubator (Thermo Fisher Scientific, Precision Model #3727, Waltham, MA, USA) was dedicated to rearing the *Wolbachia*-free (Wb^–^) colonies and it was maintained at 27 °C and a photoperiod of 12:12 (light:dark, L:D). These two colonies were fed weekly on defibrinated rabbit blood supplemented with the antibiotic rifampicin (10 µg/ml blood) and the Kao and Michayluk B Vitamin Solution (10 µl/ml blood) (Sigma-Aldrich, St. Louis, MO, USA). Note however that this vitamin solution differs substantially from the Lake & Friend [45] solution used by Hosokawa et al. [41]. Rearing vial (jar) size was increased periodically as colony numbers increased. To further mitigate external environmental contaminants, vials of blood were put under a portable UV light for 5 min immediately prior to feeding, and glass water jacketed feeders were washed with detergent and boiled for 5 min after each weekly feeding.

### Extraction of genomic DNA from *C. lectularius*

To verify that antibiotic-treated colonies of *C. lectularius* were free of *Wolbachia*, a comparison to the WS-Wb^+^ normal strain was conducted. After several filial generations, six adults were randomly selected from each of the two antibiotic-treated and vitamin supplemented colony jars (*n* = 12) and six from the untreated WS strain. Total genomic DNA was extracted using the DNeasy Blood and Tissue kit (Qiagen, Germantown, MD, USA) with a modified purification of total DNA from animal tissues (spin-column) as per manufacturer’s protocol. Individual bed bugs with heads removed (to minimize interference from eye pigments) were homogenized in the 1.5 ml microcentrifuge tube using a sterile plastic pestle and then digested overnight (~ 24 h) in 180 µl of ATL buffer solution, 20 µl of proteinase K, and 4 µl of RNase in a 56 °C water bath. Following initial digestion, samples were vortexed for 15 s, 200 µl of AL buffer was added, and then incubated in a 70 °C water bath for 10 min. After incubation, 200 µl of 96% ethanol was added, the mixture was then pipetted onto the DNeasy Mini spin column, and the DNA was bound, washed, and eluted into 200 µl of AE buffer as outlined in the protocol. An additional wash with AW2 buffer was included to further remove salts. Samples were stored at − 20 °C until polymerase chain reaction (PCR) for *Wolbachia* was conducted.

### Verification of *Wolbachia*-free *C. lectularius*

Conventional PCR was conducted to amplify a specific gene target within *Wolbachia* and measure presence or absence of *Wolbachia.* The *Wolbachia*-specific primers INTF2-FWD and INTR2-REV, adopted from Sakamoto & Rasgon [46], targeted a region of the *Wolbachia* 16S gene that produced a 136 bp amplicon. The GoTaq Green Master Mix (Promega, Madison, WI, P/N M7122) and nuclease-free water were used for all reactions at the following concentrations and volumes: 12.5 µl of 2× Master Mix, 2.5 µl of 10 µM INTF2-FWD, 2.5 µl of 10 µM INTR2-REV, 5 µl of template DNA, and PCR-grade nuclease-free water was added to achieve a final reaction volume of 25 µl. Reactions were performed using an MJ Research thermocycler (model PTC 200, Bio-Rad Laboratories, Hercules, CA, USA) with the following protocol: 95 °C for 2 min (95 °C for 30 s, 60 °C for 30 s, 72 °C for 1 min) × 36 cycles, and 72 °C for 5 min. A no-template control was used in the PCR reactions as well. A 2.0% agarose gel was used to separate the 136-bp amplicon using a 100=bp DNA ladder and GelRed nucleic acid stain (Biotium, Hayward, CA, USA), and visualized with a ChemiDoc-It TS2 imaging system (UVP, Upland, CA, USA).

Absolute quantification of *Wolbachia* in each individual bed bug was obtained with a droplet digital PCR (ddPCR) system (Model QX200, Bio-Rad Laboratories, Hercules, CA, USA) and protocol from Fisher et al. [47]. Bed bug DNA was combined with the *Wolbachia*-specific primers, TaqMan probes, and the ddPCR Supermix for Probes (Bio-Rad) into PCR-ready samples. Primers for a ribosomal protein (RPL18) specific to *C. lectularius* were used as the reference gene due to its stability [48]; they produced a 137-bp amplicon. We used double-quenched TaqMan probes with a 5′ FAM fluorophore for *Wolbachia*, a 5′ HEX fluorophore for *C. lectularius*, and 3′ Iowa Black^®^ FQ quenchers with internal ZEN quenchers (Integrated DNA Technologies, Inc., Coralville, IA, USA) specific to each target. Primer and probe sequences are listed in Table 1.

**Table 1:** *Wolbachia* and *Cimex lectularius* reference gene primer and TaqMan probe sequences

Primer/Probe	Sequence (5′–3′)
INTF2	AGTCATCATGGCCTTTATGGA
INTR2	TCATGTACTCGAGTTGCAGAGT
*Wolbachia* Probe	TGGTGTCTACAATGGGCTGCAAGG
RPL18F	GTATGACGGAGGCAGCTAGG
RPL18R	AACATTCGAGCAAATTCGGTA
*Cimex* Probe	ATGAGGACGGTGTTCTTGCCTGTC

The ddPCR reaction was optimized using extracted bed bug DNA from Wb^**+**^ and Wb^–^ lines. The bed bug/*Wolbachia* ddPCR assay comprised 22 µl of 1× Droplet Supermix (Bio-Rad), 5 µl of genomic DNA isolated from a bed bug, 2 U of *Mse*I restriction enzyme (New England Biolabs, Ipswich, MA, USA), 500 nM each of forward and reverse primers and 250 nM each of FAM- or HEX-labeled TaqMan probes for bed bug and *Wolbachia* sequences, respectively. Then the 22 µl of PCR mixtures were partitioned into an emulsion of ~ 20,000 droplets using a QX200^™^ AutoDG Droplet Digital PCR^™^ system (Bio-Rad). The PCR was performed on a T100 Thermal Cycler using the following protocol: 95 °C for 10 min and (94 °C for 30 s, 56 °C for 2 min) × 40 cycles, and 98 °C for 10 min. Post PCR, droplets were analyzed on the QX200 Droplet Reader. Absolute DNA copy numbers of bed bug and *Wolbachia* sequences in a sample were calculated on the Poisson distribution using the Quantasoft software version 1.7.4 (Bio-Rad). Previously confirmed Wb^**+**^ bed bug DNA sample and Wb^–^ bed bug DNA sample were included in each experiment as positive and negative controls. No-template control was also included in each experiment to ensure no non-specific amplifications. To estimate the limit of detection of the ddPCR assay, serial dilutions (×5, ×25, ×125, ×625, ×3125, ×15,625) of a DNA sample in water were prepared and repeated three times.

### Virus inoculations and treatments

The experiment evaluated virus titers over time in three cohorts of bed bugs: control (Wb^**+**^), antibiotic-vitamins (Wb^–^), and Wb^–^ maintained for 90 days on vitamin-supplemented blood without antibiotic. The latter (vitamin-only) group was removed from antibiotic 90 days prior to inoculation with virus.

Feline calicivirus (FCV) was chosen as the inoculum due to its environmental stability and feasibility as a viral pathogen. Feline calicivirus is a (+)ssRNA virus that is one of the primary causes of respiratory infections in felines. Virus was grown in existing Crandell Reese Feline Kidney (CrFK) cell line at the North Carolina State University College of Veterinary Medicine Clinical Virology Laboratory and stored at − 80 °C in 2 ml aliquots per established protocols. The FCV stock was produced by first removing the growth medium from confluent CrFK monolayers in 75 cm^2^ cell culture flasks by aspiration and then 1 ml of the virus inoculum was added to each flask. Flasks were incubated at 37 °C in 5% CO_2_ atmosphere for 90 min to allow for virus adsorption. Each flask then received 10 ml of maintenance medium (MEM-2% fetal bovine serum), and incubated 16 h at 37 °C in 5% CO_2_, which resulted in virus-induced destruction of ~ 90% of the monolayer.

In each feeding, 40 individual bed bugs were chosen randomly, placed in 7 ml glass vials with screened-caps, and fed as previously described for 15 min on fresh defibrinated rabbit blood supplemented with 1 ml FCV (10^7^ CCID_50_/ml) per 1 ml of blood. The CCID_50_ is the 50% cell culture infective dose, as defined below. Bed bugs were starved 7 days prior to feeding FCV-laden blood. Individuals that did not feed or only partially fed were removed and discarded. Vials were kept thereafter at room temperature under a sterile laminar flow hood and 12:12 (L:D) photoperiod.

### Quantification of FCV in *C. lectularius*

Bed bugs were killed at the following time intervals: 5 hours (h), 24 h, 4 days (d), 7 d, and 10 d post-feeding. Three randomly chosen bed bugs were removed from each cohort, sexed, and surface sterilized with 0.05% NaClO and 70% ethanol. Surface-sterilized bugs were homogenized in 3.5 ml round-bottom polystyrene tubes (Sarstedt, Nümbrecht, Germany) in 0.5 ml of Dulbecco’s Modified Eagle’s Medium (DMEM) (Caisson Laboratories, Smithfield, UT, USA) with sterile plastic pestles and the tubes were centrifuged (8000× *rpm* for 1 min). A volume of 220 µl of the supernatant was pipetted into a new 3.5 ml tube with 2.2 ml of DMEM, and serial 10-fold dilutions (10^−1^ to 10^−6^) were performed to obtain virus titration. A total of 100 µl of each dilution was placed in 4 wells (technical replicates; portrait orientation) of a flat-bottomed, 96-well plate, and 100 µl of CrFK cells was added to each well. Each plate contained a row of wells as a cell control with no virus. Plates were incubated at 37 °C for 5 d [49–51] and then stained with crystal violet (50 µl/well). FCV is a highly lytic virus; after 5 days, only wells with uninfected cells show crystal violet staining.

In each virus dilution, the percentage of dead (infected) cells was visually determined for each well. To measure the infectious virus titer, the 50% cell culture infective dose (CCID_50_) endpoint dilution assay was used to quantify the amount of virus required to kill 50% of infected CrFK cells as described by Reed & Muench [52], if the 50% dose fell between two dilutions. The Reed–Muench index formula reads as follows:

$$\frac{{\left( {\% {\text{ infected at dilution immediately above 5}}0\% } \right) - 50\% }}{{\left( {\% {\text{ infected at dilution immediately above 5}}0\% } \right) - \left( {\% {\text{ infected at dilution immediately below 5}}0\% } \right)}}$$

### Sex ratio of male and female *C. lectularius* chosen

The sex ratio of the three bed bugs randomly chosen per time interval post-feeding of FCV is shown in Table 2. Both Wb^–^ and Wb^–^ 90 d groups had close to 1:1 male:female, but slightly more males were chosen in the Wb^**+**^ group at 1:0.67 male:female ratio.

**Table 2:** Number of males and females in each bed bug group at five sampling time intervals post-feeding

Time post-feeding	Wb^+^		Wb^–^		Wb^–^ 90 d	
	M	F	M	F	M	F
5 hours	1	2	1	2	0	3
24 hours	1	2	2	1	1	2
4 days	2	1	2	1	2	1
7 days	2	1	1	2	2	1
10 days	3	0	2	1	2	1
Total	9	6	8	7	7	8

*Abbreviations:* Wb^+^, colony containing *Wolbachia*; Wb^–^, two colonies cured of *Wolbachia* with the antibiotic rifampicin; Wb^–^ 90 d, colony cured of *Wolbachia* with antibiotic, then reared for 90 days on blood supplemented with vitamins but no antibiotic; F, female; M, male

### Statistical analysis

Differences in the mean virus titer in CCID_50_/ml were analyzed with a two-sample t-test that assumed unequal variances using a 95% confidence interval with SPSS Version 19 (IBM Corp., Armonk, NY). A *P*-value < 0.05 was considered significantly different. A General Linear Model Repeated Measures analysis (Wilks’ Lambda {λ}) was also conducted in SPSS to identify any effect of time, treatment group, replicate, and interactions between these variables on virus titer.

## Results

### Confirmation of *Wolbachia*-free *Cimex lectularius* colonies

**Fig. 1:**
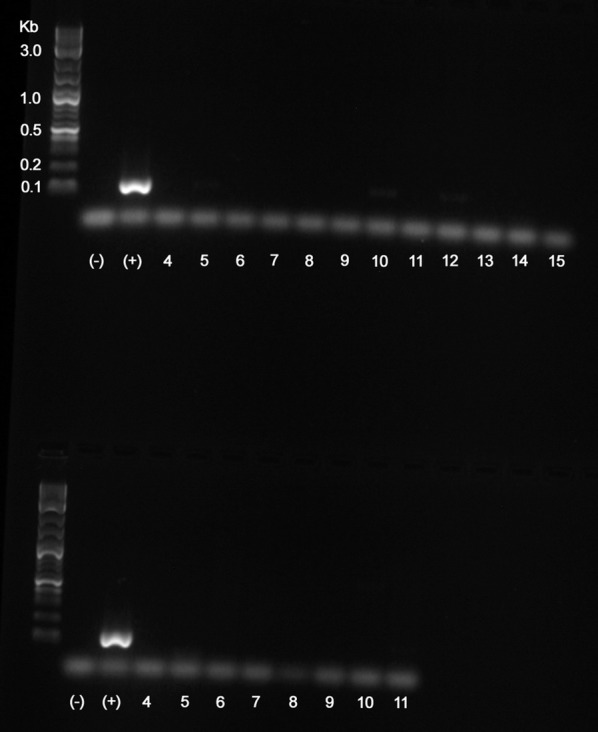
PCR results illustrating absence of *Wolbachia* in bed bugs. Top row: Lanes 4–9: bed bugs treated with the antibiotic and ingested blood supplemented with B vitamins; Lanes 10–15: bed bugs removed from antibiotics and maintained on blood supplemented with B vitamins for 30 days. Bottom row: Lanes 4–11: bed bugs removed from antibiotics and maintained on blood supplemented with B vitamins only for 60 days

The conventional PCR results confirmed that the bed bug colony treated with rifampicin and supplemented with B vitamins contained no *Wolbachia*. As well, the colony removed from antibiotics and maintained on vitamin-supplemented blood contained no *Wolbachia* (Fig. 1). The ddPCR also confirmed the absence of *Wolbachia* [47] Absolute quantification detected 0 copy numbers of the 16S *Wolbachia* target in both bed bug colonies treated with antibiotics and those later removed from antibiotics for 90 d [47]. The ddPCR was highly sensitive for detection of *Wolbachia* and bed bug DNA. Theoretical values (1.76 copies of *Wolbachia* DNA/µl; 1.00 copies of RPL18 DNA/µl) and measured values (1.40 copies of *Wolbachia* DNA/µl; 1.00 copies of RPL18 DNA/µl) matched well with high reproducibility even at extremely low concentrations (15,625-fold dilution). No *Wolbachia* or bed bug DNA was detected in the no-template controls.

### FCV acquisition and titer comparison in bed bug groups

We estimated that adult bed bugs ingested between 4-6 µl of blood meal (3.92 µl for adult males in Sierras & Schal [44]), which corresponds to 10^4^ virus in a single feeding. Live virus was detected in relatively large amounts in all treatment groups at all time intervals up to 10 d (Table 3). No significant differences were observed in FCV titer among the three treatment groups at any of the five sampling intervals, with the exception of in the Wb^**-**^ group compared to the Wb^**-**^ 90 d at the 4 d interval (*t*_(2)_ = − 4.724, *P* = 0.0179).

**Table 3:** FCV titers (log_10_ CCID_50_/0.1 ml) in each of the three bed bug groups at five sampling time intervals post-feeding

Treatment	Mean (± SE) FCV titers (log_10_ CCID_50_/0.1 ml)				
	5 hours	24 hours	4 days	7 days	10 days
Wb^+^	4.67 ± 0.00	4.56 ± 0.06	4.44 ± 0.06	3.06 ± 0.71	3.22 ± 0.49
Wb^–^	4.89 ± 0.31	4.56 ± 0.11	5.11 ± 0.11	3.50 ± 0.50	3.78 ± 0.22
Wb^–^ 90 d	4.50 ± 0.40	4.45 ± 0.00	4.17 ± 0.17	3.67 ± 0.17	3.72 ± 0.15

*Abbreviations*: Wb^+^, colony containing *Wolbachia*; Wb^–^, two colonies cured of *Wolbachia* with the antibiotic rifampicin; Wb^–^ 90 d, colony cured of *Wolbachia* with antibiotic, then reared for 90 days on blood supplemented with vitamins but no antibiotic

There was a significant decline in FCV titer over time after the blood meal (Wilks’ λ = 0.014, *F*_(2, 4)_ = 36.48, *P* = 0.0271; *ƞ*^2^ = 0.986), with an average of 90.7% decline across all three treatments after 10 d, ranging from 96.5% decline in the Wb^**+**^ group, 92.2% in the Wb^–^ group, and 83.4% decline in the Wb^–^ 90 d group (Table 3). However, there was no effect of treatment (*F*_(2, 4)_ = 0.359, *P* = 0.575) or replicate on FCV titer (*F*_(2, 8)_ = 0.127, *P* = 0.884), and there was no effect of time*treatment (Wilks’ λ = 0.072, *F*_(2, 4)_ = 6.48, *P* = 0.1381, *ƞ*^2^ = 0.928), or time*replicate (Wilks’ λ = 0.05, *F*_(4, 8)_ = 1.73, *P* = 0.3143, *ƞ*^2^ = 0.775). No infection was observed in any of the cell line controls in any of the three bed bug groups at any sampling time interval. We observed no mortality in any of the FCV-infected bed bugs that were left in the vials after feeding on FCV-supplemented blood.

## Discussion

In 1887 Elias Metschnikoff was the first to suspect that bed bugs could serve as a vector of human pathogens, but definitive proof having been elusive, several generations of researchers remained unconvinced [53]. Even today, bed bugs are not considered important vectors of any specific pathogen, although they are broadly considered medically-important due to the clinical manifestations of bite site reactions and the psychological effects that infestations can elicit [12, 54, 55]. Although more than 45 pathogens (bacteria, viruses, fungi, protozoans) have been detected in bed bugs [10], < 10% are known to replicate within the bed bug. Their apparent refractory state to human pathogens is remarkable, as nearly every other blood-feeding arthropod (mosquitoes, biting flies, lice, fleas, ticks) is associated with some type of disease or pathogen, and it may reflect the intimate and long evolutionary association of bed bugs with humans. An alternative potential human health concern is the histamine contamination that is possibly a contributing factor in allergic responses [8].

We used feline calicivirus (FCV) as a model pathogen in our investigation because it is environmentally stable, it represents RNA viruses, and it could be transmitted by bed bugs in residential settings. Since FCV is not a human pathogen, it served as a useful surrogate for other more infectious ssRNA virus without placing laboratory workers at risk of infection. Our results showed that FCV did not replicate within *C. lectularius*, but relatively high FCV titers were maintained in bed bugs 10 d after they were inoculated through a blood meal. The amount of FCV decreased dramatically over time in all of our treatment groups, from a 29% decline from 5 to 24 hours after the blood meal, to a 91% decline after 10 days. We found no evidence from these patterns that FCV could replicate within the bed bug. Although we observed a large decline in FCV, the overall decline was much smaller than other viruses evaluated in bed bugs, such as HIV and Yellow fever, where little to no replication was reported to occur [56, 57]. Importantly however, the decline in FCV titer was independent of the presence or absence of endosymbiotic *Wolbachia* (Table 3).

It is important to note that secondary effects from the rifampicin treatment could have affected the interaction of the bed bug with FCV. The *Wolbachia*-cured (Wb^–^) group was treated with antibiotic during weekly blood meals before the experiment started. Therefore, this group was also expected to suffer from an altered gut microbiome, whose possible interactions with and effects on FCV are not known. Another *Wolbachia*-cured group (Wb^–^ 90 d) was weaned off rifampicin 3 months before the experiment started. We expected this group to be less affected by the antibiotic, and its gut microbial community might have recovered during the three months, which represented less than two generations. This group, however, was not different from the other two groups, including the *Wolbachia*-containing group (Wb^**+**^) which was never exposed to rifampicin, suggesting that neither *Wolbachia* nor the gut microbiome influenced the FCV titers.

The absence of a *Wolbachia* influence on FCV titers might be related to minimal interactions between the symbiont and the virus. Factors related to the physiological conditions of the midgut, inability of FCV to permeate midgut barriers, and host immune responses may minimize these interactions. *Wolbachia*’s intracellular sequestration within the bed bug bacteriome might further diminish contact between these two microbes.

As blood is ingested, FCV would interact with a wide range of bed bug salivary proteins that are secreted to counteract the vertebrate host’s hemostasis (platelet aggregation, fibrin crosslinking, vasoconstriction, local immune responses). The bed bug genome revealed expanded families of salivary apyrases, nitric oxide carriers, and members of the Ap4a_hydrolase family [58]. Bed bug saliva contains substances that decrease ingested pathogen virulence and titers [11], but it is not known if viruses might be affected by these salivary components.

After the bed bug ingests a blood meal containing FCV, the virus must interact with the insect alimentary canal, penetrate the hemocoel, and for effective transmission with subsequent blood meals the virus needs to replicate in the salivary glands or other tissues associated with the mouthparts. Alternatively, if the virus survives passage through the alimentary canal, it can be transmitted in feces, though this pathway is considered less efficient. Mildly acidic to neutral midgut pH (5–7) is ideal for a wide range of microorganisms, but in most insects the midgut is alkaline (pH ≥ 8) and typically unfavorable for most microorganisms [59, 60]. Adult mosquito midgut pH is between 7.2–7.9 immediately prior to blood-meal ingestion, and returns to pH 7.3 after digestion [61]. The gut pH of the bed bug is not known, but if it is similar to the mosquito, it likely is reasonably favorable to FCV. Feline calici virus is a non-enveloping, environmentally stable virus, able to survive acidic to neutral pH [62]. Therefore, FCV likely survives the midgut, although the activity of digestive enzymes may hinder FCV. The bed bug genome revealed 187 potential digestive enzymes, including serine proteases, a large expansion of cathepsin D genes, and aspartic proteases that are specifically adapted for acidic pH [58].

We did not determine whether FCV was able to cross the midgut barrier and enter the bed bug hemocoel. Many insects, including blood feeders, form a peritrophic membrane (PM) around the food bolus during or shortly after ingestion. The PM is a physical barrier that protects the midgut lumen. A pathogen or parasite must penetrate the PM and invade the midgut tissues before it can cross into the hemolymph. Although the PM is absent in most Hemiptera [61], *C. lectularius*, *R. prolixus*, and *Triatoma infestans* all have a modified PM effectively known as a ‘plexiform surface coat’ type PM that is permeable to digestive enzymes [63]. The PM is one of many midgut effector mechanisms such as lectins, reactive oxygen species, nitric oxide, melanization through the prophenoloxidase cascade, and pattern recognition receptors that comprise the humoral immunity protecting the insect host against infections [64]. Several pathogens of insects escape humoral response and evade the impermeability of the PM by invading the tissues before the PM is fully developed [61]. The mosquito PM is impermeable to particles > 148 kDa [65], and most viruses are ~ 2000 kDa [66]. To permeate through the PM, viruses and other enteric pathogens must secrete proteolytic enzymes that degrade membrane proteins [66]. Once crossed into the hemolymph, most arboviruses infect all compartments of an arthropod vector [64].

If FCV crossed the PM into the hemolymph, the next host defense would be a systemic immune response by *C. lectularius* by way of hemocytes, activation of proteases, production of antimicrobial peptides, or immune signal transduction pathway activation (Toll, imd, JAK-STAT) by the fat body that could lower FCV titer. The genome of *C. lectularius* has revealed members of all these pathways, as well as the RNA interference pathway [58] and transcriptomic analysis that supports the expression of the entire suite of putative immune defense pathways [67]. It is generally thought that the combination of blood-feeding and traumatic insemination have selected for a highly adapted immune response in the bed bug. For example, the female paragenital system has an overabundance of hemocytes [55], and bed bug hemolymph and ejaculate are suspected to contain substances or “neutralizing factors” that decrease ingested pathogen virulence and titers [11]. We did not determine whether FCV was present in the bed bug hemolymph. We might speculate, however, that significant declines in the FCV titer over 10 days would suggest that FCV was attacked either in the digestive tract or in the hemolymph of the bed bug, but apparently independently of the presence of *Wolbachia*.

*Wolbachia* has been shown to play important roles in mediating host-microbe interactions. In *Drosophila*, higher *Wolbachia* densities correlate with greater antiviral protection [68, 69], and as a model for studying Blue tongue virus (BTV) replication within blood-feeding *Culicoides* midges, BTV replicated significantly in all cell lines examined from BTV-infected *Drosophila melanogaster* reared without *Wolbachia* [70]. *Wolbachia* also mediates immunocompetence in isopods [71]. In some mosquito-*Wolbachia*-virus interactions, *Wolbachia* primes the mosquito innate immune system [28, 31, 36], but there is evidence that in several mosquito species where *Wolbachia* naturally occurs, the presence of *Wolbachia* has little to no influence on resistance to or suppression of viruses [72, 73].

The localization of *Wolbachia* within the host is relevant to its involvement in pathogen suppression. In many insects, *Wolbachia* is systemically distributed either throughout the body or in specialized but highly diffuse tissues (e.g. fat body, integument). In *C. lectularius* on the other hand, *Wolbachia* resides exclusively in a bacteriome of both sexes, in association with the gonads [41] and would likely not encounter the virus to initiate a symbiont-mediated immune response. While *Wolbachia* could respond to immune challenges by remotely signaling to the fat body and hemocytes, its location in the gonad-associated bacteriome would make such a signaling pathway less likely than in mosquitoes, flies and isopods.

Bed bugs do not appear to be competent vectors for ssRNA human viruses associated with disease in humans, and their status as a medically important vector of disease remains uncertain. However, bed bugs could be more important vectors of dsDNA viruses such as hepatitis B virus (HBV). Moreover, *C. lectularius* and other cimicids conceivably may have a greater significance from a veterinary medicine perspective. Other species of cimicids may be epidemiologically important in diseases of birds and bats yet to be investigated [74]. The swallow bug *Oeciacus vicarius* can vector several arboviruses [75], and can experimentally transmit Fort Morgan virus to uninfected birds [76]. Commercial poultry operations are likely to have heavy infestations of ectoparasites [77], and the role of bed bugs as primary or bridge vectors of avian diseases is essentially unknown. Interestingly, despite an interest over the past 100 years in the potential of bed bugs to serve as vectors of human pathogens, including HIV, HBV, Ebola, Yellow Fever, Polio, Rabies, *Plasmodium*, *Leishmania*, *Yersinia*, and numerous bacterial species, immune responses by bed bugs when challenged with a pathogen remain poorly understood.

## Conclusions

To our knowledge, this is the first study to evaluate the influence of *Wolbachia* on virus titer in *Cimex lectularius*. Our results indicate that *Wolbachia* does not play a role in ssRNA virus suppression in bed bugs, in contrast to its involvement in several other hematophagous insects. These results offer further supporting evidence that bed bugs are likely not competent vectors of ssRNA viruses, adding feline calicivirus to the list of viruses examined thus far. Our conclusions underscore the need for future research to include (i) quantification of virus titers in various body compartments, particularly the hemolymph and salivary glands; (ii) hemocoel injections of virus for titer comparison in *Wolbachia*-free and normal bed bugs; and (iii) investigation of the ability of the bed bug to transmit the virus upon re-feeding.

## Data Availability

Data supporting the conclusions of this article are provided within the article. Raw datasets are available upon request.

## Abbreviations

Wb: *Wolbachia*
PCR: polymerase chain reaction
ddPCR: droplet digital polymerase chain reaction
FCV: feline calicivirus
PM: peritrophic membrane
